# Impact of Compression Stockings vs. Continuous Positive Airway Pressure on Overnight Fluid Shift and Obstructive Sleep Apnea among Patients on Hemodialysis

**DOI:** 10.3389/fmed.2017.00057

**Published:** 2017-05-19

**Authors:** Bruno C. Silva, Roberto S. S. Santos, Luciano F. Drager, Fernando M. Coelho, Rosilene M. Elias

**Affiliations:** ^1^Department of Medicine, Renal Division, Universidade de São Paulo, São Paulo, Brazil; ^2^Department of Medicine, Renal Division, Universidade Federal do Espírito Santo, Vitoria, Brazil; ^3^Instituto do Coração (InCor), Universidade de São Paulo, São Paulo, Brazil; ^4^Department of Psychobiology, Universidade Federal de São Paulo, São Paulo, Brazil; ^5^Department of Neurology and Neurosurgery, Universidade Federal de São Paulo, São Paulo, Brazil

**Keywords:** obstructive sleep apnea, compression stockings, continuous positive airway pressure, fluid shift, hemodialysis

## Abstract

**Introduction:**

Obstructive sleep apnea (OSA) is common in edematous states, notably in hemodialysis patients. In this population, overnight fluid shift can play an important role on the pathogenesis of OSA. The effect of compression stockings (CS) and continuous positive airway pressure (CPAP) on fluid shift is barely known. We compared the effects of CS and CPAP on fluid dynamics in a sample of patients with OSA in hemodialysis, through a randomized crossover study.

**Methods:**

Each participant performed polysomnography (PSG) at baseline, during CPAP titration, and after 1 week of wearing CS. Neck circumference (NC) and segmental bioelectrical impedance were done before and after PSG.

**Results:**

Fourteen patients were studied (53 ± 9 years; 57% men; body mass index 29.7 ± 6.8 kg/m^2^). Apnea–hypopnea index (AHI) decreased from 20.8 (14.2; 39.6) at baseline to 7.9 (2.8; 25.4) during CPAP titration and to 16.7 (3.5; 28.9) events/h after wearing CS (CPAP vs. baseline, *p* = 0.004; CS vs. baseline, *p* = 0.017; and CPAP vs. CS, *p* = 0.017). Nocturnal intracellular trunk water was higher after wearing CS in comparison to baseline and CPAP (*p* = 0.03). CS reduced the fluid accumulated in lower limbs during the day, although not significantly. Overnight fluid shift at baseline, CPAP, and CS was −183 ± 72, −343 ± 220, and −290 ± 213 ml, respectively (*p* = 0.006). Overnight NC increased at baseline (0.7 ± 0.4 cm), decreased after CPAP (−1.0 ± 0.4 cm), and while wearing CS (−0.4 ± 0.8 cm) (CPAP vs. baseline, *p* < 0.0001; CS vs. baseline, *p* = 0.001; CPAP vs. CS, *p* = 0.01).

**Conclusion:**

CS reduced AHI by avoiding fluid retention in the legs, favoring accumulation of water in the intracellular component of the trunk, thus avoiding fluid shift to reach the neck. CPAP improved OSA by exerting local pressure on upper airway, with no impact on fluid redistribution. CPAP performed significantly better than CS for both reduction of AHI and overnight reduction of NC. Complementary studies are needed to elucidate the mechanisms by which CPAP and CS reduce NC.

## Introduction

Obstructive sleep apnea (OSA) is a clinical condition characterized by recurrent episodes of partial or complete upper airway obstructions during sleep, promoting intermittent hypoxia and sleep fragmentation (1). The prevalence of OSA among patients with end-stage renal disease (ESRD) varies according to the diagnostic criteria used and whether it was based on questionnaires (reaching 50–80%) (2, 3) or polysomnography (PSG) (16–46%) (4, 5) and is associated with mortality among patients on hemodialysis (HD) (6, 7).

Several factors contribute to the pathophysiology of OSA in patients with ESRD, such as uremia and associated comorbidities in ESRD (8). Volume overload, which is commonly observed in these patients, plays a pivotal role in this context. It leads to edema formation in upper airways and, consequently, to pharyngeal narrowing (9, 10). Hypervolemia also augments overnight spontaneous fluid shift, which occurs as the fluid accumulated in the legs during the day moves rostrally during bedtime, while on recumbent position. This is associated with overnight increase in neck circumference (NC) and the severity of OSA, not only in patients with OSA from the general population (11) but also particularly in patients with fluid retaining states, such as congestive heart failure (CHF) (12) and ESRD (4).

Avoiding fluid accumulation in the legs during the day by wearing compression stockings (CS) is an option to attenuate the overnight fluid shift as the compression exerted increases the pressure applied to the interstitium, thus reducing fluid filtration from capillaries in the legs (13). It has been demonstrated that wearing CS during the day attenuates OSA (14–16). It is still unclear why avoiding leg swelling leads to lower obstructive respiratory events during the night. A plausible mechanism is that the hydraulic pressure exerted upward leads to fluid retention in the trunk, thus increasing daytime diuresis and, consequently, lowering total body water (TBW). The effects associated with the wearing of CS in relation to fluid redistribution and severity of OSA in oliguric and anuric patients are unknown.

On the other hand, the standard treatment for OSA, the continuous positive airway pressure (CPAP), avoids airway collapsibility, by mechanically exerting pressure on airways, thus preventing some critical anatomic points to collapse, such as the retropalatal region of the oropharynx (17). However, another consequence of such pressure is avoiding fluid accumulation in upper airways, once relatively small amounts of fluid (100–200 ml) enlarge airway soft tissues and may cause airway constriction (18). Even though CPAP may reduce such local edema, it is not clear whether such local and “downward” pressure interferes in overall body fluid shift.

Therefore, this randomized crossover clinical trial was conducted to evaluate the short-term impact of wearing CS and CPAP on the severity of OSA and fluid redistribution between legs and trunk in a sample of oliguric or anuric patients on HD. We also evaluated the impact of these interventions on overnight changes in NC. We hypothesized that CS would interfere in overnight rostral fluid shift from the legs, with consequent improvement of OSA, while CPAP would act locally on upper airways and improve OSA regardless of fluid shift.

## Materials and Methods

### Study Design

This was a randomized crossover trial that compared the effect of CS and CPAP on apnea–hypopnea index (AHI), NC, and fluid redistribution in oliguric or anuric patients on HD. OSA was screened by Berlin questionnaire and further confirmed (defined as AHI > 5 events/h) by a baseline PSG. Patients were randomly assigned for another PSG either for CPAP titration or after wearing CS for 1 week during daytime and then crossed over to the other treatment. Randomization was done by block, with two possibilities: baseline-CS-CPAP or baseline-CPAP-CS. Patients had not been previously treated with CPAP or CS before study entry. The study was approved by the local Ethics Committee for Research Project Analysis—Cappesq (#11302/2013) and registered in Clinical Trials platform (NCT02503215). All patients have signed written informed consent.

### Subjects

Patients were recruited from the HD service of the Hospital das Clínicas, Universidade de São Paulo, Brazil. Inclusion criteria were as follows: (1) dialysis vintage of at least 6 months; (2) age between 18 and 70 years; (3) diagnosis of OSA by overnight PSG; and (4) residual diuresis lower than 500 ml/day. Exclusion criteria were as follows: (1) diagnosed heart failure, atrial fibrillation, chronic obstructive pulmonary disease or neoplasia; (2) inferior limbs prosthesis or amputation; (3) current CPAP treatment; (4) negative screening by the Berlin scale; (5) thrombosis of superior vena cava; (6) presence of ascites or pleural effusion. During the study, there was no change in any prescribed medication, including antihypertensive drugs and erythropoietin-stimulating agents.

### Hemodialysis

All dialysis were performed using 4008S machines and high-flux polysulfone membranes from Fresenius Medical Care™ (Bad Homburg, Germany). The prescription was according to the attending nephrologists with no interference in the duration of the sessions and ultrafiltration rates during the study protocol. Routine exams performed regularly in our service were assessed and included. Dialysis dose, assessed by standard *Kt*/*V*, where *K* = dialyzer clearance, *t* = time, and *V* = volume of body water. We also assessed annual echocardiogram data, which provided the following variables: ejection fraction calculated by Teichholz’s formula and left ventricular mass index (LVMI).

### Berlin Questionnaire and Epworth Sleepiness Scale (ESS)

Berlin questionnaire was administered to all patients as screening for OSA (19). Patients at low risk for OSA according to this scale were excluded from study protocol. ESS (20) was applied before PSG exam in two moments: at baseline and after wearing CS for 1 week.

### Polysomnography

Overnight PSG was performed using the Embla™ S 4500 (Embla Systems, Inc., Broomfield, CO, USA) at the Sleep Laboratory of Hospital das Clínicas. Sleep stages and arousals were scored according to standard techniques (21). All subjects slept with a single pillow on a flat bed. Thoracoabdominal motion was monitored by respiratory inductance plethysmography and nasal airflow by nasal pressure cannulas (22), while arterial oxyhemoglobin saturation was continuously monitored by pulse oximetry. Obstructive apneas were defined as cessation of airflow for at least 10 s, while hypopnea was defined as a 50% or greater reduction in airflow from baseline, yet remaining above 0 for more than 10 s with thoracoabdominal motion or flow limitation on the nasal pressure tracing associated with either an oxygen desaturation of greater than 3% or an arousal. Central apneas or hypopneas were diagnosed by the absence of such thoracoabdominal movements. AHI was calculated as the total overnight number of obstructive and central apneas and hypopneas divided by total sleep time (TST). Sleep efficiency was calculated as percentage of TST of the time spent in bed after lights out. Both REM sleep and all stages of non-REM sleep were quantified as a percentage of TST. Electromyographic recordings of leg movements from the anterior tibialis muscles by standard surface electrodes allowed assessment of periodic leg movement index (PLMI). Patients were allowed to wake up freely. All sleep studies were scored by the same physician, who was blinded to the use of CS or baseline and to the measurements of leg fluid volume and NC.

Each patient underwent the baseline, and the two other patients underwent PSG exams in the same interdialytic interval, specifically in the same weekday.

### Body Fluid Volumes, Body Weight, NC, and Residual Urine Output

Segmental tetrapolar bioelectrical impedance (BIS) was performed in all patients while recumbent, before and after each PSG exam, by the multifrequency InBody™ S10 (Biospace Co., Ltd., Korea) device, which allowed assessment of the following parameters: TBW, lower limbs total water content (LLW), lower limbs extracellular water content (ecLLW), trunk total water content (TW), and trunk extracellular water content (ecTW). NC was measured above the cricothyroid cartilage with a tape measure just before bedtime and right after waking in the morning; body weight was verified in the same period. We considered “excess weight” the amount above the dry weight (defined as a routine by the dialysis team and commonly achieved at the end of dialysis session). Residual urinary output was assessed using a questionnaire, and patients should be either anuric or have no more than two episodes of diuresis per day, with an estimated volume lower than 200 ml each (<500 ml/day). Overnight variations of studied parameters were defined as delta, which represents the nocturnal variation (difference between measurements in the evening, just before the PSG and that obtained in the next morning).

### CPAP Titration

All subjects underwent PSG for CPAP titration, with ResMed S9 Escape™ (ResMed Ltd., Australia) CPAP device, which was manually titrated by the same technician to determine the optimal pressure level according AASM (23). The lowest CPAP pressure (4 cmH_2_O) was initially applied to all patients and increased progressively as needed. The optimal CPAP pressure was determined when the pressure could eliminate apnea, hypopnea, desaturation, arousals, and snoring in supine position.

### Compression Stockings

Patients were fitted with appropriately sized below-the-knee Sigvaris™ (Sigvaris Corp., Switzerland, manufactured by Sigvaris Brazil) CS, exerting an ankle pressures between 20 and 30 mmHg. Patients were instructed to wear CS for 1 week, immediately after wake up and removing them just before going to sleep. On the last day of this period, PSG was repeated.

### Outcome Measures

The primary outcome was the overnight fluid redistribution assessed in the three PSG exams. Secondary outcomes were variation of NC and change in AHI in PSG exam after CPAP titration and after wearing CS in comparison to baseline.

### Statistical Analysis

Continuous variables were expressed as mean ± SD or median (25 and 75 percentiles) according to normal or abnormal data distribution, respectively. Categorical variables were expressed as proportions. Repeated measures analyses of variance or Friedman’s test were used to compare changes in variables among baseline, CPAP, or CS exams, as appropriate. For between-group analyses, Dunn’s test was applied when null hypothesis was rejected. Statistical analyses were performed by SPSS 20 (SPSS Inc., Chicago, IL, USA) and GraphPad Prism 5 (GraphPad Software, La Jolla, CA, USA). The sample size was calculated assuming a 35% reduction in AHI after wearing CS, based on a previous randomized trial (15), with a two-tailed α of 0.05 and β of 80%. The resulting sample size was 14 patients.

## Results

### Baseline Characteristics, Enrollment, and Dialysis Parameters

**Figure 1:**
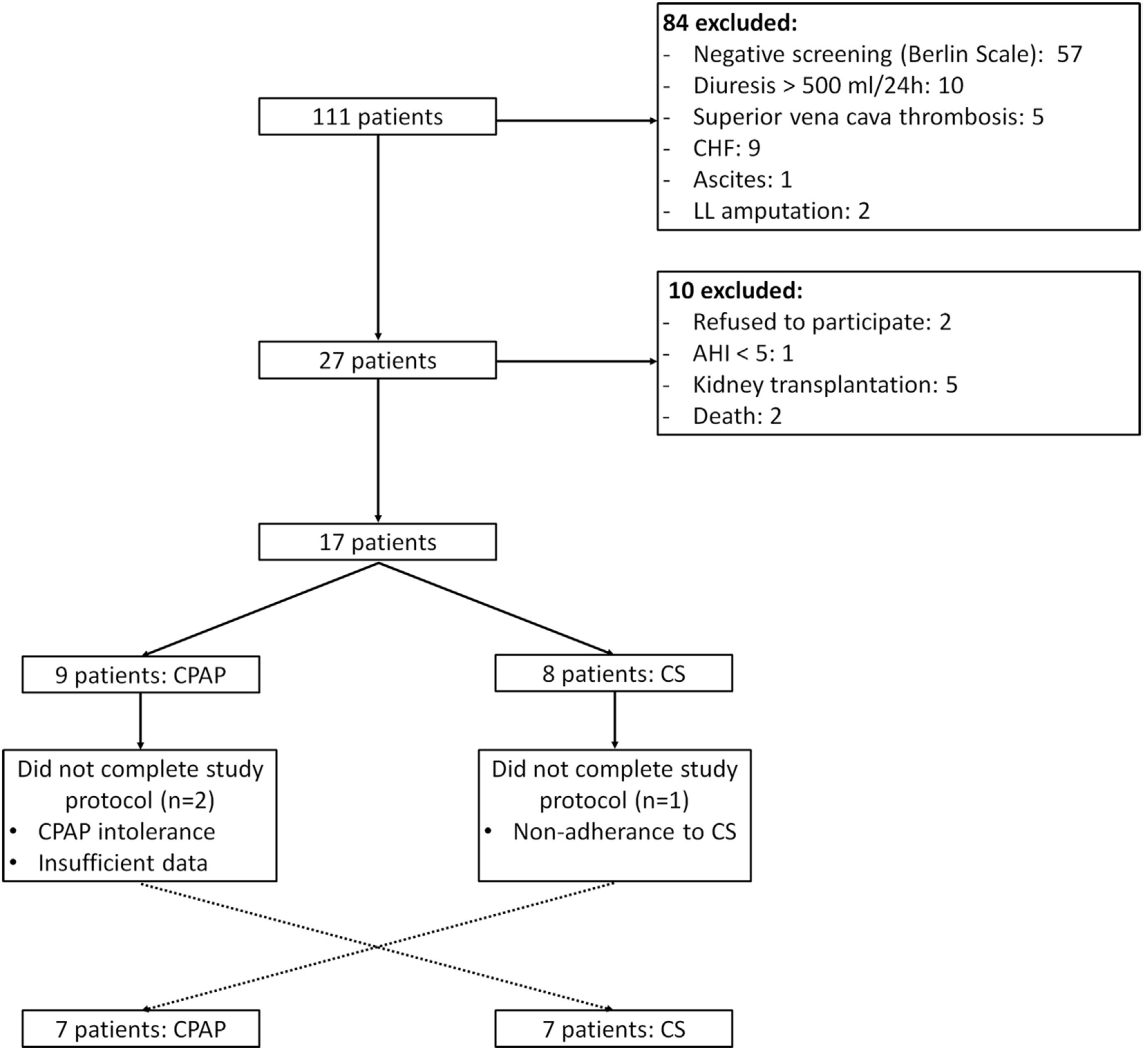
**Study flow**. CHF, congestive heart failure; LL, lower limb; AHI, apnea–hypopnea index; CS, compression stockings.

From January to December 2015, 111 patients from HD service were screened, of whom 18 underwent PSG and 14 completed the study protocol, as depicted in Figure 1. The baseline characteristics of the studied population are described in Table 1. Overall, patients were middle aged, mainly men, and Caucasian. Dialysis efficiency, assessed by standard *Kt*/*V*, was within recommended targets (24). LVMI was within normal range (<95 g/m^2^ in women and <115 g/m^2^ in men) in eight patients (57%). The majority of patients were anuric, and none of them were taking any diuretic. Time from baseline PSG to the second exam was 64 (26, 242) days and from the second to the third PSG exam was 10 (7, 21) days.

**Table 1:** **Cohort characteristics**.

Characteristic	*N* = 14
Age (years)	53 ± 9
Gender (% male)	57
Race (% Caucasian)	86
Body mass index (kg/m^2^)	29.7 ± 6.8
Dialysis vintage (months)	32 (19; 109)
Anuria (%)	64
Standard *Kt/V*	2.14 ± 0.54
Blood flow (ml/min)	350 (337; 350)
Dialyzate flow (ml/min)	714 ± 140
Dialysis duration (h/session)	4 (3.8; 4)
Arteriovenous fistula (%)	86
Arterial hypertension (%)	57
Diabetes mellitus (%)	14
Smoking habitus (%)	14
Ejection fraction (%)	64 ± 3
LVMI (g/m^2^)	119 ± 38
ACE inhibitors (%)	50
CCB (%)	21
β-Blockers (%)	21
Creatinine (mg/dl)	11.0 ± 2.9
Albumin (mg/dl)	4.15 ± 0.53
Hemoglobin (g/dl)	11.8 ± 1.0
PTH (pg/ml)	379 (311; 614)
VitD (ng/ml)	33.5 (22.7; 37.5)
Ionized calcium (mg/dl)	4.8 ± 0.3
Phosphorus (mg/dl)	5.5 ± 1.4

*LVMI, left ventricular mass index; ACE, angiotensin-converting enzyme; CCB, calcium channel blockers; VitD, vitamin D*.

*Data are presented as mean ± SD or percentage*.

### Sleep Data

Epworth sleepiness scale was performed at baseline and after wearing CS and neither changed: 10.2 ± 4.6 vs. 8.4 ± 5.2, respectively, *p* = 0.132. Table 2 shows PSG data. Patients slept on average of 5–6 h, with 75% sleep efficiency.

**Table 2:** **Sleep study data in the three moments of study**.

Sleep variables	Baseline	CPAP	Compression stockings	*p*
TST, min	307.6 ± 83.7	321.7 ± 88.7	351.9 ± 107.5	0.203
PLMI, events/h	0 (0; 0.2)	4.5 (0; 60.7)	8.9 (0; 74.2)*	0.006
N1 sleep, % of TST	10.1 (6.9; 31.1)	10.2 (6.0; 21.4)	8.6 (4.3; 15.1)	0.223
N2 sleep, % of TST	46.2 ± 8.9	43.1 ± 13.0	45.9 ± 13.8	0.528
N3 sleep, % of TST	23.3 ± 12.2	25.8 ± 15.2	22.9 ± 11.8	0.535
REM, % of TST	12.9 (8.8; 20.2)	17.6 (14.0; 19.7)	19.2 (11.3; 22.0)	0.092
Rem latency, min	115 (81.0; 175.5)	111 (85.2; 138.8)	91 (64.2; 178.5)	0.794
Arl, events/h of sleep	10.8 ± 5.8	10.7 ± 7.1	7.9 ± 4.7	0.192
WASO, min	81.6 (50.1; 176.4)	82.3 (44; 112.2)	70.3 (45.3; 111.3)	0.135
Minimal SaO_2_, %	85.0 (81.5; 87.2)	87.5 (80.7; 90.2)	83.5 (76.7; 88.0)	0.056
Mean SaO_2_, %	93.9 ± 1.9	94.8 ± 1.8^†^	93.8 ± 1.9^‡^	0.019
AHI, events/h	20.8 (14.2; 39.6)	7.9 (2.8; 25.4)^†^	16.7 (3.5; 28.9)*^‡^	0.0004
AH time, % of TST	11.2 (6.9; 17.1)	4.3 (1.4; 10.1)^†^	8.1 (2.3; 13.9)*	0.004
OAI, events/h	2.9 (1.1; 10)	0.8 (0.1; 2.9)^†^	1 (0; 7.2)	0.033
CAI, events/h	0.4 (0.1; 2.4)	0.6 (0; 1.9)	0.2 (0; 0.9)	0.941
REM AHI, events/h	11.4 (0.5; 19.4)	1.5 (0.5; 3.5)	1.8 (0; 7.1)	0.172
Supine position, %	41.6 ± 24.6	44.9 ± 30.1	48.3 ± 32.8	0.654
Supine AHI, events/h	34.1 (21.9; 50.9)	18.9 (6.5; 33.6)	33.6 (5.7; 42.0)	0.285

*AHI, apnea–hypopnea index; AH, apnea–hypopnea; Arl, arousals; WASO, wake after sleep onset; TST, total sleep time; SE, sleep efficiency; PLMI, periodic limb movement index; OAI, obstructive apnea index; CAI, central apnea index*.

*p < 0.05 CS vs. baseline.

*^†^p < 0.05 CPAP vs. baseline*.

*^‡^p < 0.05 CS vs. CPAP*.

### Effect of Interventions on AHI

**Figure 2:**
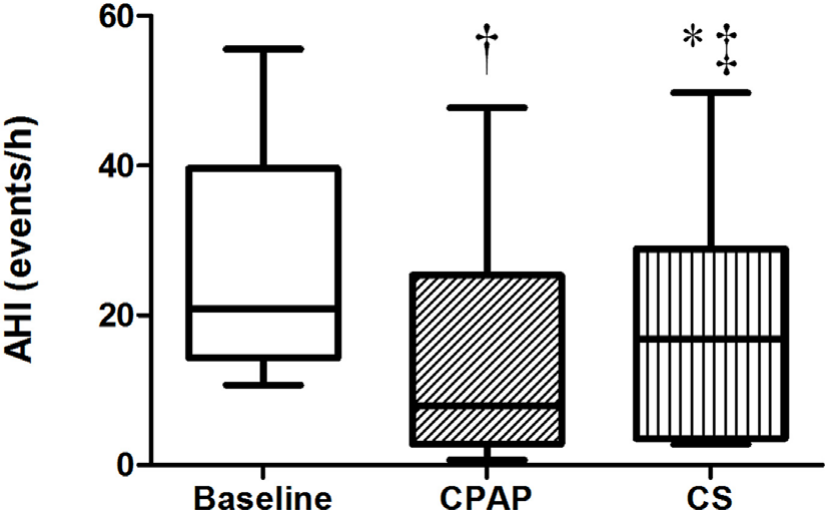
**Apnea–hypopnea index at baseline, with continuous positive airway pressure (CPAP) titration and while wearing compression stockings (CS)**. *N* = 14 for the three groups. ^†^*p* < 0.004 CPAP vs. baseline; *p = 0.017 CS vs. baseline; ^‡^*p* = 0.017 CS vs. CPAP.

The baseline AHI was 20.8 (14.2; 39.6) events/h, indicating that patients exhibited moderate OSA, and this index reduced to 7.9 (2.8; 25.4) during CPAP titration and to 16.7 (3.5; 28.9) events/h after wearing CS for 1 week (Table 2; Figure 2). CS also reduced apnea-hypopnea time but increased PLMI in comparison to baseline. When limiting our analysis only to obstructive events, CPAP reduced obstructive apnea index from 2.9 (1.1, 10) to 0.8 (0.1, 2.9) events/h, *p* = 0.021 and hypopnea index from 14 (6.8, 21.9) to 2.2 (0.9, 11.6) events/h, *p* = 0.05. CPAP also increased SaO_2_. CS did not improve any of these parameters. Central apneas were virtually absent in this population, representing 0.4 (0.1, 2.4) events/h at baseline with no changes during either CS or CPAP titration.

### Effect of Interventions on NC

**Figure 3:**
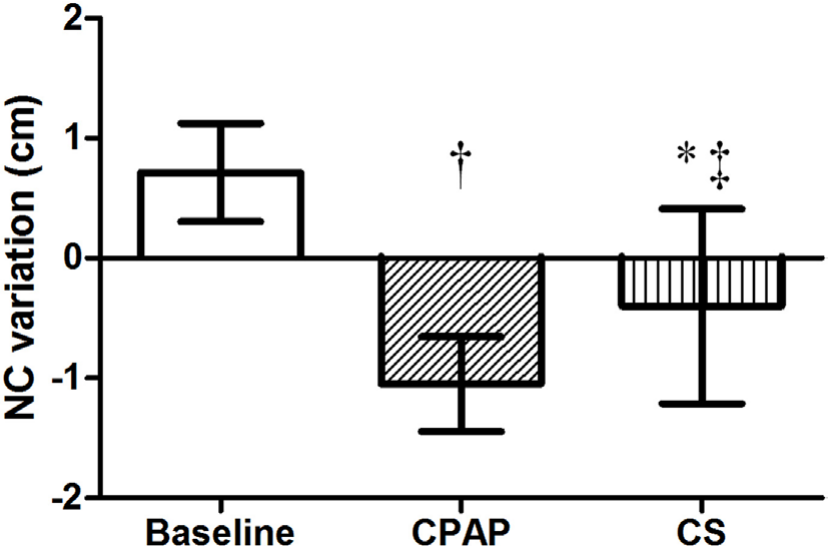
**Overnight NC variation at baseline, with CPAP titration and after wearing CS**. *N* = 14 for the three groups. CPAP, continuous positive airway pressure; CS, compression stockings; NC, neck circumference. ^†^*p* < 0.0001 CPAP vs. baseline; *p = 0.001 CS vs. baseline; ^‡^*p* = 0.01 CS vs. CPAP.

There were no differences regarding NC before sleep in the three exams; however, its overnight change presented a distinct behavior: while there was an increase at baseline, CPAP and CS promoted significant reductions in NC, as shown in Table 3 and Figure 3.

**Table 3:** **Data on weight, total, and segmental fluid volumes assessed before sleep (nocturnal) and their overnight variation (Δ) and NC, obtained at baseline and during CPAP titration and compression stockings (CS) use**.

	Baseline	CPAP	CS	*p*
Ultrafiltration, l/week	8.6 ± 2.3	8.8 ± 2.8	9.1 ± 3.0	0.524
Dry weight, kg	77.2 ± 22.6	77.8 ± 23.2	78.0 ± 23.3	0.253
Excess weight, kg	1.3 ± 1.5	1.8 ± 1.8^†^	1.8 ± 1.9*	0.028
ΔWeight, mg	−5.9 ± 54	35 ± 456	−341 ± 570	0.057
Nocturnal TBW, l	37.14 ± 7.99	37.23 ± 8.13	37.48 ± 7.81	0.740
ΔTBW, l	−0.04 ± 0.78	−0.16 ± 0.51	−0.44 ± 0.78	0.305
Nocturnal LLW, l	12.76 ± 3.18	12.87 ± 3.32	12.67 ± 3.19	0.628
Nocturnal ecLLW, % of LLW	38.5 ± 1.8	38.6 ± 2.0	38.2 ± 2.1	0.055
ΔecLLW, ml	−183 ± 72	−343 ± 220^†^	−290 ± 213	0.006
Nocturnal TW, l	17.0 ± 4.1	17.1 ± 4.1	17.5 ± 4.1*	0.030
ΔTW, ml	400 ± 464	414 ± 417	186 ± 513	0.227
Nocturnal ecTW, % of TW	38.1 ± 1.5	38.3 ± 1.5	37.9 ± 1.6^‡^	0.032
Nocturnal NC, cm	39.9 ± 4.4	40.4 ± 4.6	40.4 ± 4.8	0.150
ΔNC, cm	0.7 ± 0.4	−1.0 ± 0.4^†^	−0.4 ± 0.8*^‡^	<0.0001

*NC, neck circumference; TBW, total body water; LLW, lower limbs total water content; ecLLW, lower limbs extracellular water content; TW, trunk total water content; ecTW, trunk extracellular water content*.

*p < 0.05 CS vs. baseline.

*^†^p < 0.05 CPAP vs. baseline*.

*^‡^p < 0.05 CS vs. CPAP*.

### Effect of Interventions on Fluid Shift

Table 3 shows a comparison of the three moments of the study protocol (baseline, CPAP titration, and after wearing CS for 1 week) regarding fluid distribution. As weight and ultrafiltration are strongly associated with fluid status in patients on dialysis, data on these parameters are also shown in Table 3. Although estimated dry weight and ultrafiltration did not change during the study, excess weight (above predicted dry weight) was lower at baseline.

**Figure 4:**
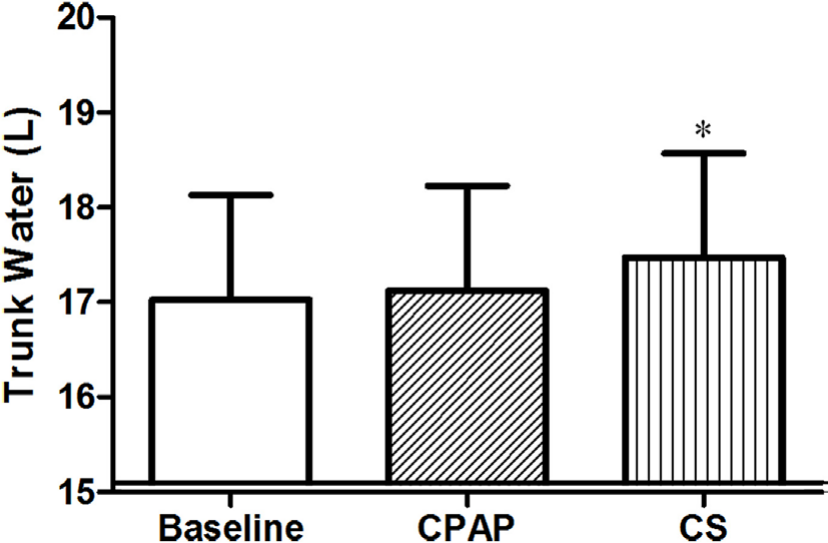
**Total trunk water volume before sleep, at baseline, before polysomnography for continuous positive airway pressure (CPAP) titration, and after wearing compression stockings (CS)**. *N* = 14 for the three groups. *p = 0.019 CS vs. baseline.

**Figure 5:**
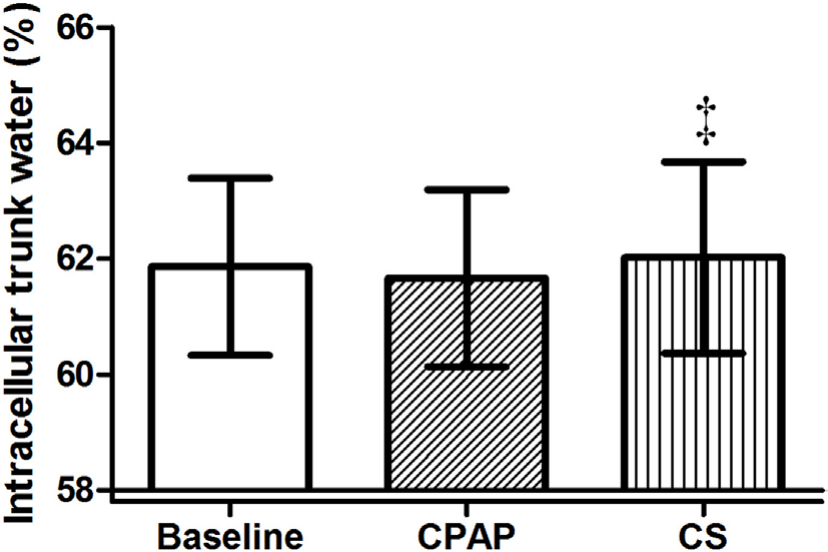
**Intracellular trunk volume (relative to total trunk water) before sleep, at baseline, before polysomnography for continuous positive airway pressure (CPAP) titration, and after wearing compression stockings (CS)**. *N* = 14 for the three groups. ^‡^*p* = 0.01 CS vs. CPAP.

Compression stockings promoted the followings changes in body fluid distribution: they increased TW before sleep compared to baseline (*p* = 0.030), which is illustrated in Figure 4; they reduced extracellular TW compared to CPAP (*p* = 0.032). As TW is equal to the sum of ecTW and intracellular trunk water, this excess fluid shifted to the intracellular compartment of the trunk (Figure 5). CPAP did not avoid extracellular volume displacement from the legs (ΔecLLW), which was even higher than at baseline.

**Figure 6:**
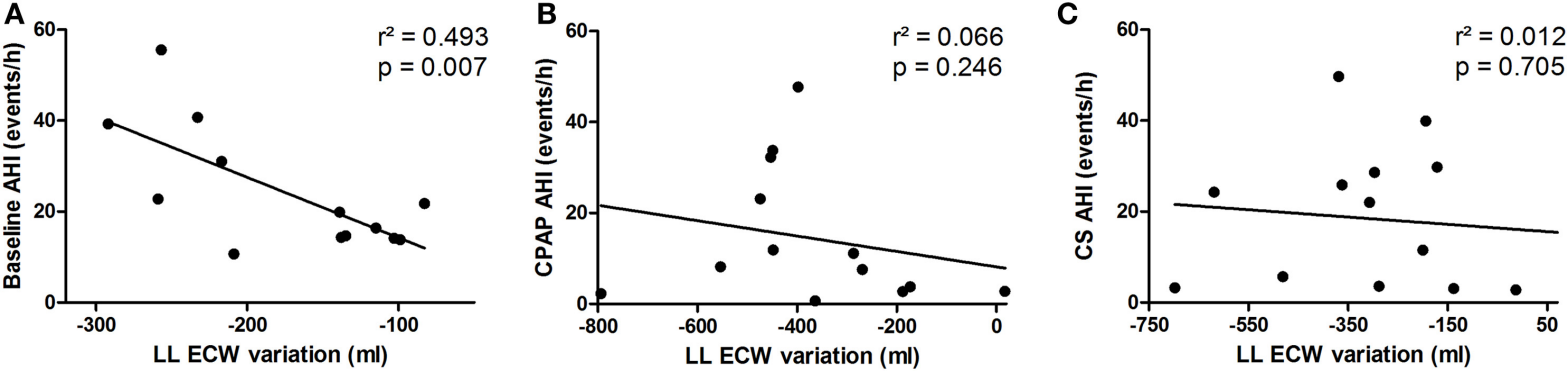
**Correlation between extracellular fluid shift from the legs (lower limbs extracellular water content) and apnea–hypopnea index (AHI) at baseline (A), continuous positive airway pressure (CPAP) titration (B) and compression stockings (CS) (C)**.

Fluid shift from the legs, represented by overnight variation of ecLLW, was only significantly associated with AHI at baseline (*r*^2^ = 0.493, *p* = 0.007), as shown in Figure 6A. Fluid shift observed during CPAP titration and after wearing CS had no significant impact on AHI (Figures 6B,C, respectively).

## Discussion

To our knowledge, this is the first study comparing overnight fluid shift and NC changes between CS and CPAP in a population on HD. We have also compared the effect of both therapies, CS and CPAP, on the severity of OSA. Our results revealed that (1) daytime use of below-the-knee CS for 1 week attenuated OSA severity in oliguric/anuric patients on HD although this effect was lower in comparison to CPAP; (2) the effects of CS were most likely attributed to displacement of fluid from the legs to the intracellular compartment of the trunk during the day, thus avoiding overnight rostral shift of extracellular fluid from the legs to the neck; (3) short-term CPAP therapy, on the other hand, did not avoid overnight fluid shift, yet it promoted a prominent reduction NC. Taken together, our findings suggest that CS may be an alternative and feasible treatment for attenuating OSA in HD patients. Furthermore, they suggest that CS and CPAP are effective on the treatment of OSA through distinct influences on overnight fluid distribution.

Obstructive sleep apnea is associated with a myriad of cardiovascular complications in the general population, such as coronary artery disease, stroke, CHF, and arterial hypertension (25–28). In patients with ESRD, who already exhibit an excessive cardiovascular risk (29), the impact of OSA can be even more devastating. Fluid overload is a possible link between high prevalence of cardiovascular disease and OSA in the setting of ESRD (30–32). Recently, hypervolemia has also been associated with more severe OSA in patients on HD (33). Indeed, there is an established background in literature in favor of fluid shift as a pathogenic factor for OSA in edematous states. In this regard, inflation of antishock trousers shifts fluid rostrally, mimicking spontaneous fluid shift and thus increasing pharyngeal resistance and narrowing the upper airway cross-sectional area in healthy individuals (34–36). In addition, magnetic resonance imaging study has shown that the severity of OSA correlates with jugular vein volume and the amount of mucosal water content surrounding the upper airway in patients on HD (37), suggesting an important role of hypervolemia in the pathogenesis of OSA in this population. In this regard, proof of concept was demonstrated by ultrafiltration without interference in uremic status in patients on HD, which attenuated OSA in this population (38).

So far, three studies have evaluated the impact of CS in individuals with preserved renal function and reported reductions in AHI from 24 to 36% (14–16). Redolfi et al. evaluated the impact of wearing CS for 1 week in patients with venous insufficiency (15), showing that despite the same body weight in both control group and after CS period, there was a 150-ml reduction in leg fluid volume after wearing CS and, more importantly, a 600-ml reduction in TBW. Therefore, less fluid was available to move rostrally, which reduced NC increase. Neither trunk volumes nor diuresis was evaluated. Nevertheless, in light of their and our findings, we can postulate that CS may avoid translocation of fluid from the trunk to the legs during the day, which increases diurnal diuresis, thus reducing TBW. In our study, patients were oliguric or anuric, and TBW was not different at baseline or after wearing CS. Still, there was a marked difference regarding fluid arrangement, once CS prevented fluid from shifting from the trunk to the legs during the day and also led to an increased intracellular TW.

To explain why intracellular TW increases after wearing CS, it is important to understand how fluid shifts according to body position. In healthy individuals, moving from recumbent to standing position, extracellular volume increases while intracellular water decreases (39). While standing up, gravitational forces exert pressure on the liquid column of the body and, consequently, hydrostatic pressure increases in the lower limbs (40). The capillary pressure in the legs when standing (90–120 cmH_2_O) exceeds the pressure needed for moving fluid to interstitial compartment (15–20 cmH_2_O) (41, 42). In addition, intravascular volume decreases 300–400 ml while standing (43, 44). Therefore, fluid shifts from intravascular and intracellular spaces, increasing extracellular volume in the lower limbs.

In this study, wearing CS prevented fluid movement from the trunk to the lower limbs and also from the intracellular to the extracellular space of the trunk. Therefore, the upward pressure exerted by CS partially counteracted gravity forces, avoiding leg swelling and keeping more fluid in the intracellular space, which is less likely to move freely to other regions of the body. Consequently, there was a reduction of the total amount of fluid reaching the neck, which partially prevented the edema buildup in upper airway during the night. This is the most likely mechanism associated with the reduction of NC and AHI while wearing CS.

Besides fluid distribution with CS, another novelty of this study was the analysis of body fluid kinetics during CPAP titration exam. We demonstrated that positive pressure exerted on upper airways had no effect on fluid shift from the legs. Still, NC reduced significantly during the night, suggesting an important reduction in upper airways edema. Therefore, regardless of excess body water volume or nocturnal fluid shift, CPAP therapy effectively abolishes obstructive events by exerting local pressure that moves fluid away from the neck, thus avoiding the upstream volume to reach upper airways, and as shown in Figures 2 and 3, CPAP performed significantly better than CS for both reduction of AHI and overnight change in NC. Finally, the mechanisms involved in the reduction of both AHI and NC by CS and CPAP are completely different: the former interferes in fluid redistribution during the night, while the latter prevents fluid reaching the neck.

This study has strengths and limitations. The main strengths were the randomized and crossover study design, as well as the use of multifrequency BIS, which allowed us to precisely estimate total, extracellular, and intracellular fluid volumes in each body segment. We also provided a novelty by demonstrating that CS can alleviate OSA in patients on HD. In addition, new insights regarding the physiology involved in the overnight fluid shift contributed to elucidate the mechanisms of action of CS and CPAP therapy. One of the limitations of this study was the small sample size, which makes the study prone for both type I and II errors. Another limitation was the non-precise assessment of residual diuresis volume, although most patients were anuric. Even though the referred urinary output was estimated (and not measured) to be lower than 200 ml per episode, this information is likely to be accurate since there is a trend toward a lower bladder reservoir in patients with long dialysis vintage (45). The NC measurement is also prone to error, although there is no gold standard method to access it. Finally, this study is a short-term clinical trial. Our main focuses were the mechanisms of fluid kinetics during the night in three different situations. To ascertain the clinical outcomes of both CPAP and CS therapies in this population, a longer treatment period for each of these treatments would be necessary. Therefore, further studies are required to evaluate adherence and long-term effect of CS and CPAP on OSA in HD patients.

In conclusion, CS and short-term CPAP can alleviate OSA in patients on HD by two different predominant mechanisms. While CS exert pressure on the lower limbs, maintaining fluid in the intracellular compartment of the trunk, CPAP therapy had no action on fluid shift, yet prevented it from reaching the neck. Even though the effect of CS is inferior to the standard CPAP treatment, CS may be an alternative and feasible treatment to attenuate OSA in HD patients. Moreover, the association of these two treatments can be useful to treat OSA and requires further investigation on ESRD patients.

## Ethics Statement

This study was carried out in accordance with the recommendations of CAPPesq (Comissão de Ética para Análises de Projetos de Pesquisa) with written informed consent from all subjects. All subjects gave written informed consent in accordance with the Declaration of Helsinki. The protocol was approved by CAPPesq.

## Author Contributions

Conception and design; analysis: BS and RE. Data acquisition: BS and RS. Interpretation of data; drafting the work or revising it critically for important intellectual content: BS, LD, FC, and RE. Final approval of the version to be published; BS, RS, LD, FC, and RE.

## Disclaimer

Financial arrangements: FAPESP (Fundação de Amparo à Pesquisa do Estado de São Paulo) provided financial support for polysomnography exams, compression stockings, and CPAP device. Non-financial arrangements: nothing to declare.

## Conflict of Interest Statement

The authors declare that the research was conducted in the absence of any commercial or financial relationships that could be construed as a potential conflict of interest.
